# The role of myeloperoxidase and myeloperoxidase–antineutrophil cytoplasmic antibodies (MPO-ANCAs) in the pathogenesis of human MPO-ANCA-associated glomerulonephritis

**DOI:** 10.1007/s10157-013-0787-7

**Published:** 2013-03-16

**Authors:** Yoshihiro Arimura, Soko Kawashima, Ken Yoshihara, Yoshinori Komagata, Shinya Kaname, Akira Yamada

**Affiliations:** First Department of Internal Medicine (Nephrology and Rheumatology), Kyorin University School of Medicine, 6-20-2 Shinkawa, Mitaka, Tokyo 181-8611 Japan

**Keywords:** MPO-ANCA-associated glomerulonephritis, Extracellular MPO, MPO-positive cells, In situ immune complex

## Abstract

It is well known that antineutrophil cytoplasmic antibodies (ANCAs) are pathogenic and have a diagnostic value for ANCA-associated vasculitis. We demonstrated that a rise in myeloperoxidase (MPO)-ANCA titers during remission is often predictive of a future relapse in MPO-ANCA-associated vasculitis. Pathological examination of renal biopsies indicated that not only MPO-ANCAs, but also extracellular MPO, an in situ immune complex composed of MPO and MPO antibodies, may play important roles in the pathogenesis of glomerular capillary injury in MPO-ANCA-associated vasculitis.

## Introduction

Myeloperoxidase-antineutrophil cytoplasmic antibodies (MPO-ANCAs) have been thought to be related to the pathogenesis of MPO-ANCA-associated glomerulonephritis (GN) by binding to the MPO molecules that appear on the surface of primed neutrophils which causes release of oxygen radicals [1]. Recent studies suggest that MPO, MPO-ANCAs, neutrophils and immune complexes may relate to the pathogenesis of MPO-ANCA-associated GN [2–10]. Here, we review our data regarding the role of MPO-ANCAs, neutrophils (MPO-ANCA-positive cells), MPO, immunoglobulins and complements in the pathogenesis of MPO-ANCA-associated GN.

## MPO release from neutrophils and sensitivity to formyl-methionyl-leucyl-phenylalanine (FMLP)

The release of MPO from neutrophils in patients with MPO-ANCA-associated GN was higher than that in healthy controls. The sensitivity of MPO release to FMLP of neutrophils in patients with MPO-ANCA-associated GN was significantly higher than in patients whose GN was not associated with MPO-ANCA and in healthy controls [2].

## Serum MPO and serum cytokines in MPO-ANCA-associated GN

Serum MPO was detected in patients with MPO-ANCA-associated GN and the amounts of MPO were especially high in the cellular crescent stage and correlated with MPO-ANCA [3]. Tumor necrosis factor-alpha and interleukin (IL)-6 were also detected in the sera in parallel with disease activity and MPO-ANCA titers [3]. IL-8 was also increased in the active stage of MPO-ANCA-associated GN [4].

## Relationship between rise in MPO-ANCA titer during remission and relapse

In 143 patients with MPO-ANCA-associated vasculitis admitted to Kyorin University Hospital from 1989−2010, 29 cases relapsed (relapse rate 20 %). The average time to first relapse after remission induction was 1.6 years. Twenty-four out of 29 patients had serial ANCA titers measured before the relapse; eighteen out of the 24 patients (75 %) relapsed after rising MPO-ANCA titers.

## Relationship between MPO-positive cells and MPO on the glomerular capillary wall

**Fig. 1:**
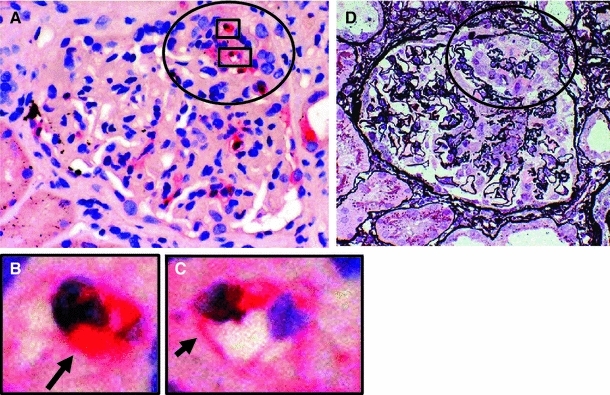
MPO staining in the glomeruli of patients with MPO-ANCA-associated glomerulonephritis. **a** MPO-positive cells and MPO are shown in the glomerulus and along the glomerular capillary wall, respectively. **b** MPO in the cytoplasm of a polymorphonuclear leukocyte (*arrow*) (MPO staining). **c** MPO along the glomerular capillary wall (*arrow*) (MPO staining). **d** Periodic acid silver methenamine and hematoxylin and eoxin staining on the serial sections in active segmental necrotizing glomerular changes

**Fig. 2:**
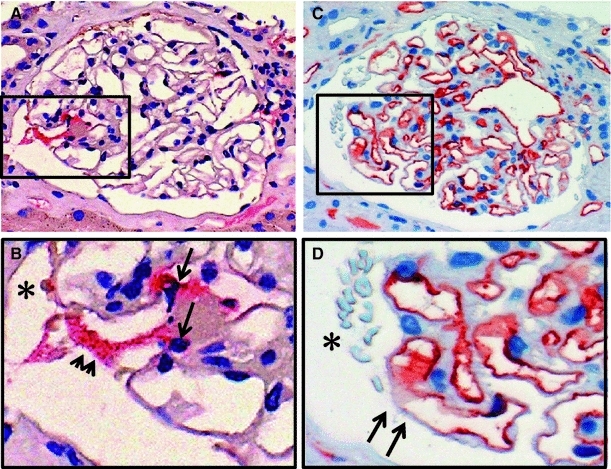
Comparison of MPO and CD34 staining on the serial sections in early segmental change glomerulus. **a**–**c** MPO staining: MPO (*red*), nucleus (*blue*). MPO-positive cells (*long arrows*) are observed in the glomerular capillary lumen. MPO is stained along the glomerular capillary walls (*short arrows*) near the MPO-positive cells. **c**, **d** CD34 staining: CD34 (*red*), nucleus (*blue*). CD34 staining decreased (*arrows*) on the glomerular capillary wall. Red blood cells (*asterisk*) are observed in the Bowman’s space, which suggesting the rupture of the glomerular capillary wall

MPO existed along the glomerular capillary walls near the infiltrated MPO-positive cells in active (Fig. 1a–c) and early-phase necrotizing GN (NGN) (Fig. 2a, b). CD34 staining was decreased on the adjacent area of the same glomerulus (Fig. 2c, d). Many MPO-positive cells and MPO along the glomerular capillary wall were detected in active and more severely damaged NGN (data not shown) [5]. MPO-positive cells and MPO were not detected on the glomerular capillaries during inactive and chronic-phase NGN [5].

## Double immunofluorescence staining (MPO and CD34)

**Fig. 3:**
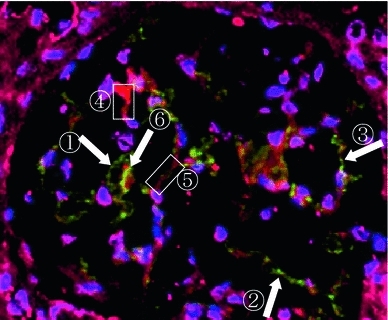
Double staining of MPO and CD34 by immunofluorescence microscopy. ①②③: *Green* shows MPO-positive staining. MPO is stained along the glomerular capillary wall without CD34 staining. ④⑤: *Red* shows CD34-positive staining. CD34 is stained along the glomerular capillary wall without MPO staining. ⑥: *Yellow* shows double-positive staining of MPO and CD34. *Blue* shows nuclear cell

MPO was detected along the glomerular capillary wall near MPO-positive cells which was accompanied by decreased staining of CD34 in some areas of the glomerulus suggesting capillary injuries (Fig. 3). In other areas, double staining of MPO and CD34 was seen [5, 6].

## Triple immunofluorescence staining (MPO, immunoglobulin (Ig) G and CD34)

IgG was associated with MPO along the CD34-negative glomerular capillary walls but was also detected alone in other areas near the capillaries [5, 6].

## Relationship between C3, IgG and MPO on the glomerular capillary wall

MPO, IgG and C3 staining was seen on the same area during the early stage of GN [6].

## Conclusion

We demonstrated that serum MPO, MPO release, and sensitivity to FMLP from neutrophils increased in patients with MPO-ANCA-associated GN [2, 3]. Clinically, a rise in MPO-ANCA titers during remission was often predictive of a future relapse in MPO-ANCA-associated vasculitis. Histological examination showed many MPO-positive cells and MPO along the glomerular capillary wall in early-phase and in more active and severely damaged MPO-ANCA-associated NGN. MPO, IgG and C3 deposition on the same area was detected mainly during the early phase [6], suggesting that immune complexes containing MPO may be pathogenetically important especially in the early phase of the disease. Recently, Kessenblock et al. [7] reported that neutrophil extracellular traps, which contained MPO and nuclear fragments in the chromatin fibers and are released from ANCA-stimulated neutrophils, result in glomerular capillary necrosis in ANCA-associated GN. We concluded that extracellular MPO released from activated MPO-positive cells, and in situ immune complexes composed of MPO and MPO antibody, may play a pathogenic role in glomerular capillary injury in the early stage of MPO-ANCA-associated NGN.
